# Detection of *Treponema pallidum* DNA by targeting the *tp0574* and *tp0548* genes in genital lesion, oral swab, and anal swab samples from a cohort of Peruvian patients with syphilis

**DOI:** 10.1128/spectrum.01809-25

**Published:** 2026-02-26

**Authors:** Francesca Vásquez, Maria Eguiluz, Silver K. Vargas, Jazmin Qquellon, E. Michael Reyes-Diaz, Lorenzo Giacani, Carlos F. Caceres, Jeffrey D. Klausner, Kelika A. Konda

**Affiliations:** 1 Centro de Investigación Interdisciplinaria en Sexualidad, Sida y Sociedad, Universidad Peruana Cayetano Heredia, Lima, Perú; 2 Department of Medicine, Division of Allergy and Infectious Diseases, University of Washington 7284, Seattle, Washington, USA; 3 Department of Global Health, University of Washington 7284, Seattle, Washington, USA; 4 Department of Population and Public Health Sciences, Keck School of Medicine, University of Southern California 5116, Los Angeles, California, USA; Ascension St John Hospital, Detroit, Michigan, USA

**Keywords:** *Treponema pallidum*, syphilis, PCR, *tp0574*, *tp0548*, Peru, *T. pallidum*, extragenital, lesion

## Abstract

*Treponema pallidum* (*T. pallidum*) can be detected by molecular techniques. However, research is needed to determine the best specimen and DNA gene target for this molecular detection. Previous research has employed the *tp0574* gene as a target in nucleic acid tests for *T. pallidum* detection, whereas the *tp0548* gene has mainly been used for molecular typing of *T. pallidum*. We evaluated the effectiveness of using *tp0574* alone and both targets, *tp0574* and *tp0548*, for *T. pallidum* detection in anal, oral, and lesion swab samples from syphilis cases. Between 2019 and 2022, participants diagnosed with syphilis in Peru were enrolled. A total of 258 oral, 116 anal, and 87 lesion swab samples were collected and processed to amplify both target genes using qualitative PCR. *T. pallidum* DNA was detected in 8.5% oral, 38.8% anal, and 49.4% lesion samples. We obtained a significantly higher proportion of positives when both genes were used compared with using *tp0574* alone. Detection rates increased from 3.9% using *tp0574* only to 8.5% when combining both *tp0574* and *tp0548* in oral samples (*P* < 0.05), and detection in lesion samples increased from 24.1 to 49.4% using both targets (*P* < 0.001). We also observed significant differences when comparing the proportion of participants with *T. pallidum* DNA detected between those with one and two or three samples collected *(P* < 0.001). Amplifying *tp0574* and *tp0548* genes improves the detection of *T. pallidum* DNA, and collecting samples from multiple anatomical sites (anal and oral samples) could facilitate syphilis diagnosis when lesions are not observed.

**IMPORTANCE:**

*Treponema pallidum* DNA amplification may be a useful technique to aid in the diagnosis of syphilis and increase case infection. While specific genes have been used in PCR techniques for this purpose, the *tp0574* gene has been used as a main target due to its specificity; the best single or combination of gene targets is unknown. Given the *tp0548* gene is employed for genotyping, only a few studies have evaluated its utility for *T. pallidum* detection. Here, we have evaluated two gene targets (*tp0574* and *tp0548*) for *T. pallidum* DNA detection in extragenital and lesion samples from persons with syphilis. We found that the implementation of *tp0548* as a second target improves *T. pallidum* DNA detection and that tp0548 alone performs better than tp0574 in both oral and lesion samples. However, further research is necessary to explore the implementation of tp0548 target to improve the detection in diverse types of samples.

## OBSERVATION

Syphilis is a sexually transmitted infection (STI) caused by the spirochete bacterium *Treponema pallidum* subspecies *pallidum* (*T. pallidum*). Despite effective treatment, syphilis continues to cause significant morbidity and mortality worldwide. The World Health Organization (WHO) estimated 8 million new cases of syphilis worldwide in 2022 (1). Key populations, such as men who have sex with men (MSM) and transgender women (TW), experience high rates of syphilis (2, 3). In Peru, the most recent report estimated about 40–55% of those populations had a history of syphilis (4) and were frequently co-infected with HIV (5).

Symptomatic syphilis is more common in early stages. Primary syphilis typically presents with a chancre (or primary lesion) in the genital areas, while secondary syphilis is more commonly associated with a rash rather than a localized chancre (5, 6). Approximately 5% of primary lesions are located in extragenital areas, and these are sometimes misdiagnosed due to their atypical presentations (7–9). Moreover, other STIs, such as herpes simplex virus (HSV) and *Haemophilus ducreyi* (chancroid), can also produce genital lesions similar to those seen in syphilis (10, 11). Syphilis diagnosis is commonly based on serological tests that include treponemal and lipoidal (non-treponemal) antigen tests. Syphilis diagnosis can be challenging, as treponemal antibodies persist throughout the patient’s life, even after treatment; this persistence makes treponemal tests inappropriate for assessing reinfections or confirming a successful treatment response. Additionally, treponemal tests can only detect syphilis antibodies a few weeks after exposure, which may result in false-negative results in recently infected patients (11–13). The implementation and use of molecular detection via nucleic acid amplification tests (NAATs) are becoming more widespread in clinical and research practices (14–17). Considering the ability of *T. pallidum* to invade organs and tissues, it is logical to enhance existing diagnostic methods using molecular approaches to facilitate early detection in lesion, oral, vaginal, and anal samples (6).

Techniques based on polymerase chain reaction (PCR) are useful tools in syphilis diagnosis and research (16). Although PCR tests are confirmatory methods for primary syphilis diagnosis recommended by the Centers for Disease Control and Prevention, further research is needed to determine the best specimen and target type for molecular detection (18). Past studies have shown that *T. pallidum* DNA can be found in saliva and in oral and anal cavities (17, 19, 20). The *tp0574* gene (also called *tpp47*) is a commonly used target for *T. pallidum* detection in biological samples (16, 21, 22). Another common amplification target, a short region of the *tp0548* gene, encoding a periplasmic membrane protein with FadL-like orthologs (23), is mainly used for *T. pallidum* genotyping due to its variability (24). However, few studies have used this gene for *T. pallidum* DNA detection. Here, given the well-established *tp0548* amplification protocol, we evaluated the *tp0574* and *tp0548* genes as molecular targets for *T. pallidum* DNA detection in anal, oral, and lesion samples from syphilis cases to see if using both targets together can improve detection.

We enrolled participants diagnosed with clinical syphilis at public HIV/STI clinics in Peru. Individuals who had syphilis were enrolled between May 2019 and June 2022 as part of the Picasso study (25). Participants had follow-up visits to ensure treatment success and then to continue syphilis screening. Overall, we enrolled 264 participants with a new syphilis diagnosis. We also found repeat cases of syphilis during follow-up visits. A repeat infection was defined in those with new syphilitic lesions or a fourfold rise in non-treponemal (lipoidal) titer and a history of successful treatment defined as a ≥4 fold RPR titer decrease compared to the prior titer at initial diagnosis. Clinical staging of syphilis was determined by trained health clinicians following WHO guidelines. All clinical staging was verified by experienced syphilis investigators, and any uncertain cases were reviewed and resolved through consensus between study researchers and site staff. Early syphilis was defined based on either clinical finding (e.g., presence of a chancre or rash consistent with primary and secondary syphilis, respectively) or a serological criterion (≥4 fold increase in RPR titer within the past year). Both clinical and laboratory pieces of evidence were used to assign the appropriate stage. Asymptomatic cases who met serological criteria of early syphilis were diagnosed as early latent syphilis. If the criteria for early syphilis were not met, patients were diagnosed with syphilis infection of unknown duration.

Rapid point-of-care tests were used in each clinic to assess participants for syphilis (Alere Determine Syphilis TP, Alere Inc., USA). All individuals were screened using a non-treponemal (lipoidal) test, the rapid plasma reagin (RPR) test (Wiener, Argentina). The *Treponema* pallidum particle agglutination (TPPA) test (Serodia, Fujirebio Diagnostics, Inc., Japan) was used for confirmatory syphilis testing.

We collected oral and anal swabs for *T. pallidum* screening from enrolled participants, including those with re-infections (anal swab collection was initiated in November 2020; therefore, we obtained a smaller number of anal samples). Participants were examined by a clinician, and a lesion swab was collected if a lesion was present. “Sample type” is defined as the anatomical site from which the swab was taken: oral, anal, or lesion. All swab samples were collected in 500 µL of lysis buffer (1 M Tris pH 8.0, 0.5 M EDTA, 10% SDS) and then transported frozen to the Laboratory of Sexual Health at Universidad Peruana Cayetano Heredia in Lima, where they were kept at −20°C until DNA extraction.

DNA was extracted from all swab samples using Quick DNA kits (Zymo Research, USA) according to the manufacturer’s instructions and then amplified by qualitative PCR using GoTaq DNA Polymerase (Promega, USA). Amplification targeted the *tp0574* and *tp0548* genes for *T. pallidum* detection. Human DNA control was included in each PCR reaction to monitor for potential nonspecific amplification. In addition, the human *β-globin* gene was amplified as internal extraction control. Protocols for PCR amplification and all primer sequences were previously described in the literature (26–28). Detailed protocols and additional data can be found in Tables S1 to S3 in the Supplementary Material.

For statistical analysis, we estimated the proportions of samples with *T. pallidum* DNA detected. A two-sample proportion test was applied to assess whether the detection rate was significantly different when using either target (tp0574 or tp0548) compared to the *tp0574* target alone for each sample type (oral, anal, or lesion). We also compared the proportion of *T. pallidum* DNA detected in individuals with one, two, and three sample types using the two-sample proportion tests. In addition, we calculated the proportion of *T. pallidum* DNA detection by sample type using the *tp0574* and *tp0548* gene targets alone or in combination. Stata SE v.17 (College Station, TX) software was used for all analyses.

Among all the 264 syphilis cases, 81 (30.7%) were classified as primary, 23 (8.7%) as secondary, 137 (51.9%) as early latent, and 23 (8.7%) as latent infection of unknown duration. Most cases (233; 88.3%) had an initial RPR titer of ≥1:8. In addition, 149 (56.4%) individuals had a history of past syphilis (confirmed by a laboratory record), and nearly half (42.4%) were living with HIV.

Overall, 258 oral, 116 anal, and 87 lesion swab samples were collected. *T. pallidum* DNA (using *tp0574* or *tp0548* target) was detected in 22/258 (8.5%) oral swabs, 45/116 (38.8%) anal swabs, and 43/87 (49.4%) lesion swabs, regardless of syphilis stage. The total proportion of participants who had a positive result for *T. pallidum* DNA defined as successful amplification of either the tp0574 or tp0548 target was 98/260 (37.7%).

*T. pallidum* DNA detection using *tp0574* and *tp0548* targets by anatomical region was, respectively, 3.9 and 5.0% from oral swabs, 38.8 and 15.5% from anal swabs, and 24.1 and 47.7% from lesions swabs. In Table 1, we compare *T. pallidum* DNA detection using *tp0574* alone versus using either *tp0574* or *tp0548*. Notably, treponemal DNA detection in oral samples 8.5% was higher when we used either *tp0574* or *tp0548* target compared to using only the *tp0574* target 3.9%, *P* < 0.05. Likewise, detection in lesion samples also improved when both targets were used (49.4%) compared to using only the *tp0574* target (24.1%), *P* < 0.001. As shown in Table S4 in the supplementary material, we conducted an exploratory analysis of *T. pallidum* DNA detection by each PCR target and sample type across syphilis clinical stages. While this analysis suggests that detection rates varied across stages and sample types, especially with increased positivity in lesion samples when combining both gene targets (*tp0574* or *tp0548*), sample sizes within some strata were small, and this analysis was, therefore, included for descriptive purposes only.

**TABLE 1:** *T. pallidum* DNA detected by target in conventional PCR assay^*b*^

Sample type	*n*	*T. pallidum* DNA detection				*P* value
		*tp0574*		*tp0574* or *tp0548*^*a*^		
		Pos *n* (%)	95% CI	Pos *n* (%)	95% CI	
Oral	258	10 (3.9)	1.5–6.2	22 (8.5)	5.1–11.9	0.014
Anal	116	45 (38.8)	38.8–45.2	45 (38.8)	38.8–45.2	0.500
Lesion	87	21 (24.1)	15.2–33.1	43 (49.4)	38.9–59.9	<0.001

^*a*^ Amplification of either *tp0574* or *tp0548* genes in the same sample.

^*b*^ Pos, positive.

Among the 264 participants enrolled, four did not have any swab samples (oral, anal, or lesion) collected and were, therefore, excluded from the sample-based analysis. Of the remaining 260 participants, 91 provided one sample type (90 only oral sample, one only anal); 137 provided two sample types (82 oral and anal, 54 both oral and lesion, and one both lesion and anal samples); and 32 provided all three sample types. Among participants who provided all three sample types, *T. pallidum* DNA was detected in 20/32 individuals (62.5%). For participants with two samples collected, 69/137 (50.4%) had at least one positive sample. Among participants who had only one sample type collected, 9/91 individuals (9.9%) had a positive result. We observed differences when comparing the proportions of participants with *T. pallidum* DNA detected between those with one and two samples collected (*P* < 0.001) and between those with one and three samples collected (*P* < 0.001).

**Fig 1:**
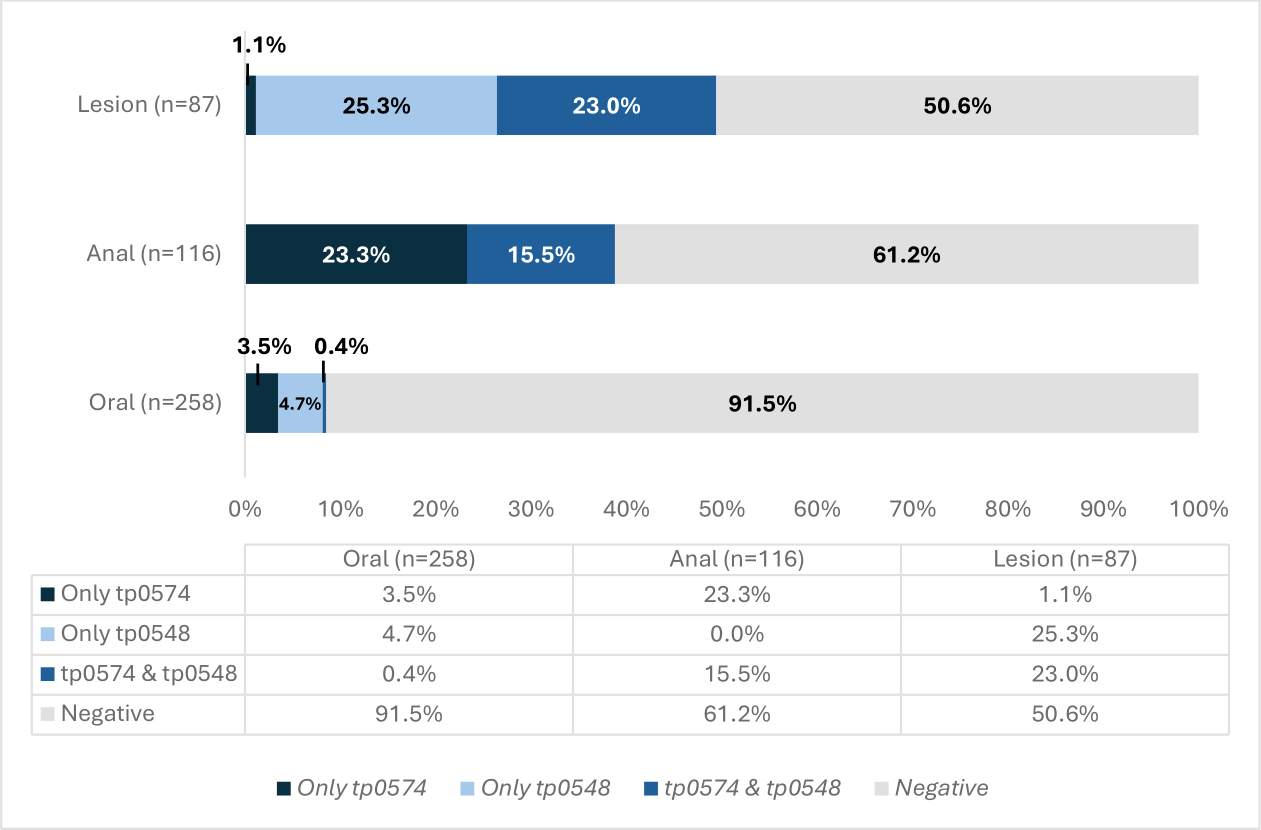
Distribution of *T. pallidum* targets positivity among syphilis patients by sample type. *tp0574* & *tp0548* indicate simultaneous amplification of *tp0574* and *tp0548* at the same time in the sample.

In Fig. 1, we illustrate the distribution of positivity of *tp0574* and *tp0548* alone or in combination to detect *T. pallidum* DNA by sample type (oral, anal, or lesion). Among the oral samples, 3.5% were positive to only *tp0574*; 4.7% were positive to only *tp0548*; and 0.4% were positive to both targets at the same time (*tp0574 & tp0548*). In anal samples, 23.3% were positive to only *tp0574*, and 15.5% were positive to both targets at the same time (*tp0574 & tp0548*); no positives were detected using *tp0548* alone. Finally, among lesion samples, 1.1% were positive to only *tp0574*; 25.3% were positive to only *tp0548*; and 23.0% were positive to both targets at the same time (*tp0574 & tp0548*).

In the present study, we successfully detected *T. pallidum* DNA in samples from the anal cavity (38.8%), the oral cavity (8.5%), and chancre lesions (49.4%) by using these gene targets alone or in combination. These results highlight the potential utility of using anal and oral swab samples for syphilis detection in agreement with previous studies (5, 25–27). Although some studies have reported a high rate of *T. pallidum* DNA in oral samples without visible chancre lesions and have also demonstrated the presence of treponemal DNA in the saliva of syphilis patients (17, 28, 29), we observed a lower detection of *T. pallidum* DNA in oral samples compared to those studies. The differences may be due to the sample collection method whereby oral rinse may be more sensitive than oral swabbing (30).

In our study, we have demonstrated the utility of using both *tp0574* and *tp0548* target genes to enhance *T. pallidum* DNA detection overall and the potential use of the *tp0548* target alone in oral and lesion samples. A higher proportion of *T. pallidum* DNA in oral and lesion samples was detected by using both targets in combination (*tp0574* or *tp0548*) compared with using only the *tp0574* target, demonstrating the possibility of including *tp0548* as a second target to improve *T. pallidum* detection. Although there is no established consensus on the specific targets recommended for *T. pallidum* DNA detection, the *tp0574* and *polA* genes are among the most commonly utilized targets due to their high concordance in detection accuracy (15, 31).

Although our results showed the possibility to use *tp0548* alone as a target for *T. pallidum* detection, it by itself does not perform well in anal samples, where *tp0574* detected most of the *T. pallidum* DNA-positive specimens. We detected a higher number of positive results when using both *tp0574* and *tp0548* targets in combination in the same sample type, particularly in oral samples at various stages and in lesion samples from primary syphilis cases. This supports the usefulness of using multiple DNA targets to enhance detection sensitivity. Even though the proportion of positive samples using the *tp0574* target was higher than using the *tp0548* target in anal samples, the additional use of *tp0548* target in oral and lesion samples improved detection. Our findings support the utility of collecting multiple anatomical samples to enhance *T. pallidum* detection, which may be particularly important when lesions are not present or sampling is performed at asymptomatic sites (32).

Our study does have limitations, as our sample population included a low number of cisgender women; further studies may be necessary to confirm if our results obtained for *T. pallidum* detection would similarly apply. Additionally, using an oral rinse might improve detection of *T. pallidum* from the oral cavity (30).

In conclusion, our work highlights some interesting findings: using both *tp0574* and *tp0548* gene targets in combination improved the detection of *T. pallidum* DNA in patients with syphilis, including those in the primary, secondary, and early latent stages. Additionally, we have demonstrated the difference in using those targets for *T. pallidum* detection across different anatomical sites. Further studies are needed to clarify these target-specific performance differences and establish the best target in specific sample types.

## SUPPLEMENTAL MATERIAL

Supplemental material spectrum.01809-25-s0001.docx Tables S1 to S4.

ASM does not own the copyrights to Supplemental Material that may be linked to, or accessed through, an article. The authors have granted ASM a non-exclusive, world-wide license to publish the Supplemental Material files. Please contact the corresponding author directly for reuse.
