# Supplementary material for: Detection of Treponema pallidum DNA by targeting the tp0574 and tp0548 genes in genital lesion, oral swab, and anal swab samples from a cohort of Peruvian patients with syphilis
Source: Microbiol Spectr. 2026 Feb 26;14(4):e01809-25. doi: 10.1128/spectrum.01809-25 (PMC13055379; doi:10.1128/spectrum.01809-25)
Supplement: Supplemental material — Tables S1 to S4. [file spectrum.01809-25-s0001.docx]

Table S1. Primers used for *T. pallidum* amplification

| **Target** | **Primer name** | **Primer sequence** | **Size (bp*)** |
| --- | --- | --- | --- |
|  |  |  |  |
| *tp0574* | tp0574_F  tp0574_R | 5’-CGTGTGGTATCAACTATGG-3’  5’-TCAACCGTGTACTCAGTGC-3’ | 310 |
| *tp0548* | tp0548_F  tp0548_R | 5’-GGTCCCTATGATATCGTGTTCG-3’  5’-CGTTTCGGTGTGTGAGTCAT-3’ | 300 |
| *b-globin* | b-globin_F  b-globin_R | 5'-GAAGAGCCAAGGACAGGTAC-3'  5'-CAACTTCATCCACGTTCACC-3' | 300 |

**bp: base pair*

Table S2. PCR protocol for *tp0548* amplification

| **Reagent** | **Volume (µl)** |
| --- | --- |
| NFW | 9.8 |
| 5X buffer GoTaq flexi  dNTPs | 4  1.6 |
| MgCl_2_  tp0548_F | 1.2  0.6 |
| tp0548_R  DNA  Taq DNA polymerase | 0.6  2  0.2 |

NFW: nuclease free water

Table S3. PCR protocol for *tp0574* amplification

| **Reagent** | **Volume (µl)** |
| --- | --- |
| NFW | 10.33 |
| 5X buffer GoTaq flexi  dNTPs | 4  1.6 |
| MgCl_2_  tp0574_F | 1.2  0.32 |
| tp0574_R  DNA  Taq DNA polymerase | 0.32  2  0.23 |

NFW: nuclease free water

Table S4. *T. pallidum* **DNA detected by target in conventional PCR assay**

| **Syphilis diagnosis** | **Sample type** | **n** | ***T. pallidum* DNA detection** | | | | ***p value*** |
| --- | --- | --- | --- | --- | --- | --- | --- |
|  |  |  | ***tp0574*** | | ***tp0574 or tp0548^a^*** | |  |
|  |  |  | **Pos**  **n (%)** | **95% CI** | **Pos**  **n (%)** | **95% CI** |  |
| **Primary** | Oral  Anal  Lesion | 80  33  79 | 2 (2.5)  11 (33.3)  19 (24.1) | -0.9 – 5.9  16.9 – 49.0  14.5 – 33.4 | 6 (7.5)  11 (33.3)  40 (50.6) | 1.7 – 13.2  16.9 – 49.0  39.9 – 62.0 | 0.073  0.500  0.000 |
|  |  |  |  |  |  |  |  |
| **Secondary** | Oral  Anal  Lesion | 22  11  6 | 4 (18.2)  7 (63.6)  2 (33.3) | 1.9 – 34.0  34.4 – 91.5  -4.6 – 7.0 | 6 (27.3)  7 (63.6)  3 (50.0) | 8.4 – 45.5  34.4 – 91.5  9.9 – 90.0 | 0.237  0.500  0.275 |
|  |  |  |  |  |  |  |  |
| **Early latent** | Oral  Anal  Lesion | 133  2 | 3 (2.3)  23 (37.1)  0 (0.0) | -0.2 – 4.8  28.7 – 45.2 | 8 (6.0)  23 (37.1)  0 (0.0) | 1.9 – 10.0  28.7 – 45.2 | 0.065  0.500 |
|  |  |  |  |  |  |  |  |
| **Latent of unknown duration** | Oral | 23 | 1 (4.4) | 1.4 – 6.5 | 2 (8.7) | -3.0 – 19.0 | 0.185 |
|  | Anal  Lesion | 10  0 | 4 (40.0)  0 (0.0) | 0.9 – 70.3 | 4 (40.0)  0 (0.0) | 0.9 – 70.3 | 0.500 |

Pos: Positive

^a^ Amplification of either *tp0574* or *tp0548* genes in the same sample
